# Comprehensive Assessment of Health Risks Associated with Gram-Negative Bacterial Contamination on Healthcare Personnel Gowns in Clinical Settings

**DOI:** 10.3390/microorganisms13071687

**Published:** 2025-07-18

**Authors:** Daniela Moreno-Torres, Carlos Alberto Jiménez-Zamarripa, Sandy Mariel Munguía-Mogo, Claudia Camelia Calzada-Mendoza, Clemente Cruz-Cruz, Emilio Mariano Durán-Manuel, Antonio Gutiérrez-Ramírez, Graciela Castro-Escarpulli, Madeleine Edith Vélez-Cruz, Oscar Sosa-Hernández, Araceli Rojas-Bernabé, Beatriz Leal-Escobar, Omar Agni García-Hernández, Enzo Vásquez-Jiménez, Gustavo Esteban Lugo-Zamudio, María Concepción Tamayo-Ordóñez, Yahaira de Jesús Tamayo-Ordóñez, Dulce Milagros Razo Blanco-Hernández, Benito Hernández-Castellanos, Julio César Castañeda-Ortega, Marianela Paredes-Mendoza, Miguel Ángel Loyola-Cruz, Juan Manuel Bello-López

**Affiliations:** 1 Hospital Juárez de México, Mexico City 07760, Mexico; 2 Sección de Estudios de Posgrado e Investigación, Escuela Superior de Medicina, Instituto Politécnico Nacional, Mexico City 11340, Mexico; 3 Hospital Psiquiátrico Dr. Samuel Ramírez Moreno, Valle de Chalco Solidaridad 56619, Mexico; 4 Escuela Nacional de Ciencias Biológicas, Instituto Politécnico Nacional, Mexico City 11340, Mexico; 5 Facultad de Medicina, Universidad Nacional Autónoma de México, Mexico City 54090, Mexico; 6 Facultad de Ciencias Químicas, Universidad Autónoma de Coahuila, Saltillo 25280, Mexico; 7 Centro de Biotecnología Genómica, Instituto Politécnico Nacional, Reynosa 88710, Mexico; 8 Facultad de Biología, Universidad Veracruzana, Xalapa 91090, Mexico; 9 División de Tecnología Ambiental, Universidad Tecnológica de Nezahualcóyotl, Nezahualcóyotl 57000, Mexico

**Keywords:** healthcare-associated infections, Gram-negative, ESKAPE bacteria, healthcare personnel gowns, infection control

## Abstract

Microbiological contamination of healthcare workers’ gowns represents a critical risk for the transmission of healthcare-associated infections (HAIs). Despite their use as protective equipment, gowns can act as reservoirs of antibiotic-resistant bacteria, favouring the spread of pathogens between healthcare workers and patients. The presence of these resistant bacteria on healthcare workers’ gowns highlights the urgent need to address this risk as part of infection control strategies. The aim of this work was to assess the microbiological risks associated with the contamination of healthcare staff gowns with Gram-negative bacteria, including the ESKAPE group, and their relationship with antimicrobial resistance. An observational, cross-sectional, prospective study was conducted in 321 hospital workers. The imprinting technique was used to quantify the bacterial load on the gowns, followed by bacterial identification by MALDI-TOF mass spectrometry. In addition, antimicrobial resistance profiles were analysed, and tests for carbapenemases and BLEE production were performed. The ERIC-PCR technique was also used for molecular analysis of *Pantoea eucrina* clones. Several Gram-negative bacteria were identified, including bacteria of the ESKAPE group. The rate of microbiological contamination of the gowns was 61.05% with no association with the sex of the healthcare personnel. It was observed that critical areas of the hospital, such as intensive care units and operating theatres, showed contamination by medically important bacteria. In addition, some strains of *P. eucrina* showed resistance to carbapenemics and cephalosporins. ERIC-PCR analysis of *P. eucrina* isolates showed genetic heterogeneity, indicating absence of clonal dissemination. Healthcare personnel gowns are a significant reservoir of pathogenic bacteria, especially in critical areas of Hospital Juárez de México. It is essential to implement infection control strategies that include improving the cleaning and laundering of gowns and ideally eliminating them from clothing to reduce the risk of transmission of nosocomial infections.

## 1. Introduction

Historically, white gowns have been considered a symbol of authority, respect, cleanliness, neatness, commitment to health, and perceived patient safety [1,2,3,4]. In particular, the use of the white coat in healthcare dates to the late 19th century, where Joseph Lister encouraged its adoption as part of antiseptic practices during surgical procedures and thus promoting its use as a symbol of hygiene, safety, and purity [5,6]. Since then, white clothing has been an integral part of the image of healthcare professionals, including nurses, orderlies, laboratory assistants, and laboratory personnel; particularly, the white coat for medical staff has become more relevant because of its impact on the microbiological safety of the patient. Such is the concern about this issue, that its traditional symbol has begun to be questioned [7,8,9]. In this context, microbiological research, involving bacterial culture of the textiles of healthcare workers’ gowns, has shown that far from being protective and safety barriers, they can act as reservoirs of pathogenic and opportunistic microorganisms, contributing to the cross-transmission of bacteria between healthcare workers and patients, leading to the emergence of healthcare-associated infections (HAIs) [7,10].

From a microbiological perspective, in 2017, Gram-negative bacteria, primarily those of the ESKAPE group, classically defined as *Enterococcus faecium*, *Staphylococcus aureus*, *Klebsiella pneumoniae*, *Acinetobacter baumannii*, *Pseudomonas aeruginosa*, and *Enterobacter* species, were recognized by the World Health Organization (WHO) as the leading causes of HAIs worldwide. However, a recent report in 2024 has included a broader range of clinically relevant genera, such as *Escherichia coli*, *Salmonella Typhi*, *Shigella*, *Citrobacter*, *Proteus*, *Serratia*, and *Morganella*. Therefore, in the critical priority list, members of the order *Enterobacterales* have been mandatorily included due to their increasing role in nosocomial infections and antimicrobial resistance mainly to fourth-generation cephalosporins and carbapenems, which can now be functionally integrated into the ESKAPE acronym, extending their epidemiological relevance beyond the original six pathogens [11,12].

As can be seen, this acronym has been dynamic in its microbiological composition. In this regard, De Rosa et al. highlighted that the ESKAPE group was never intended to be a closed list but rather a conceptual representation of organisms that “escape” the effects of antimicrobial agents due to their resistance mechanisms and adaptability in clinical settings [13]. Furthermore, debates within the scientific community have emphasized that the ESKAPE group, although originally defined as a fixed set of pathogens, should be interpreted as flexible and expandable, rather than a rigid acronym [14]. This evolving perspective reflects the growing recognition that other bacteria exhibit resistance mechanisms equivalent to those of classical ESKAPE members, including the production of ESBLs and carbapenemases. Several of these genera have been implicated in hospital outbreaks and clonal dissemination events. Therefore, the ESKAPE group should be interpreted as a flexible and expandable framework, rather than a rigid acronym. This allows for its adaptation to the changing landscape of antimicrobial resistance while recognizing that even less common but resistant *Enterobacterales*, although not always considered primary agents of HAIs, can serve as critical reservoirs of resistance determinants in healthcare settings.

ESKAPE group bacteria are not only relevant because they are the responsible for HAIs but also because they have the ability to acquire antimicrobial resistance mechanisms, such as plasmids, transposons, and integrons, which translates into difficult eradication to antimicrobial treatment [15]. In other studies, on pathways of transmission of in-hospital pathogens, healthcare staff gowns were identified as potentially contaminated by *K. pneumoniae*, a carbapenemase-producing ESKAPE member, after handling patients infected with this microorganism [16]. Such is the importance of this problem, that contamination of staff hands by *A. baumannii* has been found to be associated with an increased likelihood of contamination of staff gowns [17]. This highlights the possible relationship between contamination of healthcare staff gowns and the occurrence of HAIs and outbreaks in critical units. In the local context, in our working group, the presence of ESKAPE bacteria has been documented in different scenarios, such as contaminants of invasive medical devices, hands of health personnel, clonal dispersion of ESKAPE bacteria causing outbreaks in the COVID-19 period, and generators of collapse of the pulmonary microbiota, among others [18,19,20,21]. These findings not only demonstrate the problem of these bacteria in the hospital environment but also their possible persistence, where the gowns of healthcare personnel could play an important role in the chain of transmission of pathogens to the patient, as they are often inadvertent routes of transmission. It is important to note that the microbiological risk is not limited to antibiotic-resistant ESKAPE bacteria, as in patients with comorbidities and immunocompromised patients, such as those with cancer, HIV/AIDS, transplant recipients, or chronic diseases, bacteria considered commensal or opportunistic can cause severe infections such as pneumonia, bacteraemia, and sepsis [22,23,24,25].

The problem is exacerbated among healthcare staff in training: students, interns, trainees, and nurses in clinical practice, as these staff, often with less experience and training in infection control, often make prolonged and inappropriate use of gowns, wearing them outside the clinical setting and without regular laundering. This makes gowns potential vehicles for the spread of microorganisms. Therefore, the aim of this study was to perform a risk analysis associated with the microbiological contamination of the gowns of healthcare personnel at Hospital Juárez de México (HJM), with emphasis on Gram-negative ESKAPE bacteria and other Gram-negative bacteria. This was conducted through the phenotypic and genetic characterisation of the microorganisms identified from a focus on antimicrobial resistance to carbapenemics and cephalosporins. Implications for the presence of ESKAPE group bacteria and other Gram-negative bacteria on healthcare workers’ gowns and the associated risks are analysed and discussed.

## 2. Materials and Methods

### 2.1. Temporal–Spatial Location, Study Population

This study was observational, cross-sectional, and prospective and conducted from March to May 2025. A population of 321 unduplicated healthcare workers was selected from HJM, a tertiary-level hospital located north of Mexico City. Three inclusion criteria were considered for participants: use of long-sleeved white polyester and cotton gowns (70%/30%) at the time of analysis, participation on Fridays (considering that the gown was used throughout the week), and at 12:00 h, as this was the busiest time in the hospital. Microbiological monitoring was carried out in situ, i.e., in the workplace. Six areas were selected (a, b, c, d, e, and f), since most of the hospital services are concentrated there. In addition, a population of 91 participants was recruited consisting of medical students.

### 2.2. Quantification of Gram-Negative Bacteria on Healthcare Personnel Gowns

For the quantification of Gram-negative bacteria on the gowns of healthcare personnel, the areas that frequently come into contact with patients and are considered potential sources of cross-contamination were selected. The imprinting technique was used for the quantification of colony-forming units for each back cuff of both sleeves of the gown on Mac Conkey agar plates during 10 s of contact. This procedure was performed on an area of 28 cm^2^ for each cuff. The plates were incubated at 35 ± 2 °C for 24 and 48 h and were counted and reported as CFU/28 cm^2^. The selection of morphotypes of interest was based on microbiological criteria, such as appearance, colour, size, and use of lactose as a carbon source.

Morphotypes that were not of clinical interest were eliminated and not considered for further analysis. With the information obtained, an association analysis was performed by using contingency Tables (Chi^2^) and Mann–Whitney analysis (non-parametric Student’s *t*-test) to identify significant differences in bacterial loads in both cuffs of the gowns by sex. Conversely, ANOVA and Tukey’s post hoc test were performed to determine significant differences in bacterial loads by areas of analysis. A value of *p* < 0.05 was considered for the identification of significant differences between microbiological loads by sex and areas. For taxonomic assignment of the bacteria of interest, the typical colonial morphotypes for this type of bacteria were identified and were purified in LB-agar and grown in LB-broth then frozen in glycerol (50%) and stored at −70 °C for future experiments.

### 2.3. Bacterial Identification by Mass Spectrometry MALDI-TOF

Gram-negative isolates were identified to genus and species level by the direct analysis of whole bacterial cells using matrix-assisted laser desorption/ionization-time of flight mass spectrometry (MALDI-TOF MS). For this purpose, all strains were streaked in LB-agar and incubated overnight at 37 °C, and single colonies were subjected to identification by using a Bruker MALDI Biotyper (Bruker Daltonik, Bremen, Germany) according to the manufacturer’s instructions. The criterion to best match with the identification protocol was bacterial strains with score values above 2.0 (down to 3) for high-confidence identification. MBT Compass Library version 10.0 was used to assign genus and species to the isolates. With the microbiological identification data, graphs of taxonomic diversity and relative abundance by care areas were generated, and an Alluvial diagram was generated in RAW Graph 2.0.1 to know the distribution of the ESKAPE group by healthcare area of the health personnel [26].

### 2.4. Antimicrobial Resistance Profiles

Isolates that were classified within the ESKAPE group were tested for antimicrobial resistance as follows. The antimicrobial resistance profile was determined through the guidelines established by CLSI, 2024 according to the sensitivity discs set by *Enterobacterales*, *Acinetobacter* spp., and *P. aeruginosa* [27]. The classification of resistance level was conducted according to Magiorakos et al. [28]. *P. aeruginosa* ATCC 27853 and *E. coli* ATCC 25922 were used as controls. Results were inferred as susceptible, intermediate, or resistant by measuring the diameter of the inhibition zone. The frequency of antibiotic resistance was calculated and represented in percentages (%).

### 2.5. Carbapenemases and BLEE Production and Their Relationship with Genotypes

Carbapenem-resistant bacteria strains were subjected to the modified carbapenem inactivation method (mCIM) according to Pierce et al. [29]. Extended-spectrum β-lactamases (BLEE) were detected in solid media, using discs of ceftazidime (30 μg), cefotaxime (30 μg), ceftazidime/acid clavulanic (30 μg/10 μg), and cefotaxime (30 μg/10 μg) according to CLSI [27]. *Klebsiella pneumoniae* carrying the *bla_NDM-1_* gene and *K. pneumoniae* ATCC 700603 were used as positive controls of carbapenemases [19] and BLEE producers, respectively. Finally, *E. coli* 25922 was used as a negative control.

### 2.6. Screening to Detect Resistance Genes

For carbapenemases and BLEE-producing strains, end-point PCR assays were performed to detect metallo-β-lactamases (*bla_NDM_*, *bla_VIM_*, and *bla_IMP_*), serine β-lactamases (*bla_KPC_*, *bla_OXA-48_*, *bla_OXA-23_*, and *bla_OXA-40_*), and BLEE (*bla_SHV_*, and *bla_CTX-1_*) genes using the primers shown in Table 1. Amplicons were run in 1 × TBE buffer, separated via horizontal electrophoresis in 2.0% agarose gels, visualized, compared with an appropriate molecular weight marker, and photographed under UV illumination. Controls for carbapenemases were obtained from Cortés-Ortíz et al. [19] and Loyola-Cruz et al. [20]; *K. pneumoniae* ATCC 700603 and *E. coli* BAA-3287 were used as *bla_SHV-18_* and *bla_CTX-1_* controls.

### 2.7. Molecular Typing of Pantoea eucrina by ERIC-PCR

*Pantoea eucrina* strains were subjected to genomic DNA extraction with the commercial Favorgen^®^ Genomic DNA Kit (Omega BIO-TEC, Norcross, GA, USA) according to the manufacturer’s instructions and quantified by fluorometry using the Qubit 4 Fluorometer (Thermo Fisher Scientific, Waltham, MA, USA). Additionally, they were visualised on horizontal 0.8% agarose gels. Molecular typing by ERIC-PCR was performed according to Versalovic et al. [32] as follows. The reaction volume was 50 μL and consisted of Molecular Biology grade water, 1 × PCR buffer, 20 nM MgCl_2_, 25 mM deoxyribonucleotide phosphate, 100 pM of each primer, 3 units *Taq* DNA polymerase, and 300 ng of template DNA. Cycling conditions were as follows: pre-denaturation at 95 °C for 7 min, denaturation at 90 °C for 30 s, annealing at 58 °C for 1 min, and extension at 65 °C for 8 min, with a final extension at 68 °C for 16 min at the end for 30 cycles. Genetic profiles were run 1 × TBE buffer, pH 8.3, and separated on horizontal electrophoresis in 1% agarose gels, visualised, and photographed under UV illumination.

Genetic profiles were analysed by intra-gel pattern comparison by using ImageLab 5.2.1. To confirm the reproducibility of assays, they were performed three times. Tenover criteria were used to establish the clonal relationship between isolates [33]. Finally, graphical relationship was performed through distance matrix by using a linear semilogarithmic method. The dendrograms were constructed by using the UPGMA algorithm, with the Dice similarity index. Genomic similarities were confirmed with a bootstrap test of 1000 repetitions by using the Past4 program (Version 4.09).

### 2.8. Risk Assessment by Using a Vester Matrix

With the microbiological and genetic findings, a Vester matrix was constructed to classify according to their degree of causality the problems associated with the contaminated gowns of the health personnel of HJM. Twenty-two risk scenarios were identified that could have an impact on various infectious health-risk outcomes. The causality of the scenarios was assessed individually and as a group by various health professionals, including 5 microbiologists, 3 infectious disease physicians, 3 epidemiologists, and 3 nurses. With the causality results (0, 1, 2, and 3), the most frequently occurring problems were considered to have the closest causality to reality and were transferred to the matrix to categorise them as active, passive, critical, or indifferent problems [34].

## 3. Results

### 3.1. Study Population and Related Hospital Services

Table 2 shows the study population categorised according to their professional role within the HJM. A total of 321 participants were recruited, distributed in 143 males and 178 females, respectively. The participants were distributed among 28 different hospital services, 8 of which were considered critical areas (adult, neonatal and paediatric intensive care units, operating theatres, peritoneal dialysis, haemodialysis, pneumology, and toxicology). The study population was distributed as resident physicians (48.9%), trainees (48.6%), and only 2.5%, which corresponded to assigned physicians. The group of medical students recruited (*n* = 91) consisted of 41 males and 50 females, respectively.

### 3.2. Analysis of Microbiological Contamination According to Sex

**Figure 1:**
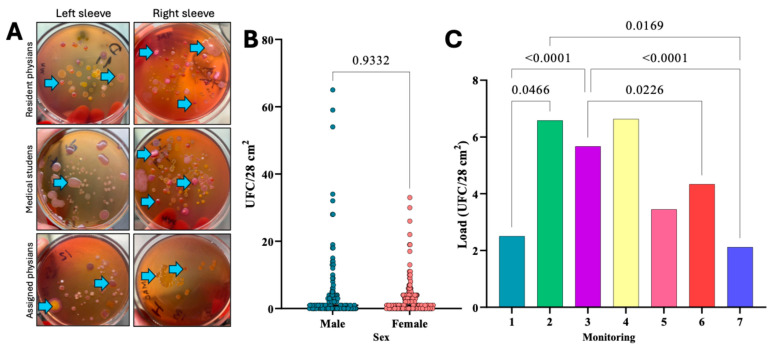
(**A**) Representative microbiological findings of Gram-negative bacterial cultures by imprinting from the back cuffs of the gown sleeves of healthcare personnel at Hospital Juárez de México. (**B**) Mann–Whitney analysis of Gram-negative microbiological load by sex and (**C**) Post hoc ANOVA analysis comparing Gram-negative bacterial loads (CFU/28 cm^2^) by hospital care areas of HJM. Correspondence between monitoring numbers (1–7) and hospital care areas is detailed in Table 2.

Microbiological analysis by imprinting of the back cuff of healthcare personnel revealed the presence of various morphotypes of clinical interest. Figure 1A shows representative images of microbiological findings with morphotypes of clinical interest and marked with blue arrows. Frequency analysis of microbiological contamination by Gram-negative bacteria showed that 87 (60.8%) and 109 (61.2%) male and female participants, respectively, showed evidence of contamination by Gram-negative bacteria, with maximum and minimum values of 60 and 37 CFU/28 cm^2^, respectively. The overall contamination rate was 61.05%, representing 196 participants. With this finding, a Chi-squared test (*p* ≤ 0.05) was performed, which showed that the sex of the health personnel is not associated with the degree of microbiological contamination by Gram-negative bacteria (*p* = 0.942). To validate the above observations, two additional tests were performed to determine the association. The first was the likelihood ratio, which showed no association between sex and bacterial growth (*p* = 1). A continuity correlation (*p* = 0.942) was performed, which showed that the observed frequencies were the same as expected, concluding that sex had no apparent effect on bacterial growth. The minimum number of samples required for the Chi-square test (*n* ≥ 5) was also exceeded. Likewise, the Mann–Whitney analysis confirmed the absence of association with the sex of health personnel (*p* = 0.9332) (Figure 1B).

### 3.3. Comparison of Microbiological Contamination of Gowns by Hospital Care Area

The results of the post hoc ANOVA test to compare the Gram-negative bacterial load (CFU/28 cm^2^) by hospital care areas, including the medical student population (monitoring 1), are shown in Figure 1C. The results show that some areas showed significant differences compared to others, suggesting important variations in the bacterial load of interest (*p ≤* 0.05). The key areas with the highest bacterial contamination were 2 (general surgery, otorhinolaryngology, adult intensive care unit, neurosurgery, endoscopy, pneumology, operating rooms, obstetrics, and gynaecology), 3 (gastroenterology, infectology, geriatrics, neurology, toxicology, orthopaedics, ophthalmology, traumatology, and urology) and 4 (internal medicine, haematology, cardiology, rheumatology, nephrology, peritoneal, and haemodialysis). Figure 1C shows the results of the post hoc ANOVA analysis comparing the Gram-negative bacterial load (CFU/28 cm^2^) by the care areas of HJM.

### 3.4. Taxonomic Characterization of Microorganisms Isolated from Healthcare Gowns

Mass spectrometry analysis of the taxonomic diversity at genus and species level of the 184 isolates from the gowns revealed the presence of a total of 12 bacterial genera. Interestingly, the yeast genus and species *Candida albicans* was detected in the microbiological isolation (monitoring 1, 3, and 7). The analysis of number of species per bacterial genus was represented as follows: *Pseudomonas* (*n* = 13), *Acinetobacter* (*n* = 7), *Pantoea* (*n* = 5), *Enterobacter* (*n* = 2), *Raoultella* (*n* = 2), *Mixta* (*n* = 2), *Serratia* (*n* = 2), *Agrobacterium* (*n* = 1), *Citrobacter* (*n* = 1), *Erwinia* (*n* = 1), *Ochrobactrum* (*n* = 1), and *Pseudoscherichia* (*n* = 1). Analysis by focus area revealed the presence of 7, 15, 7, 16, 11, 22, and 7 different species in monitoring/areas 1, 2, 3, 4, 5, 6, and 7, respectively.

**Figure 2:**
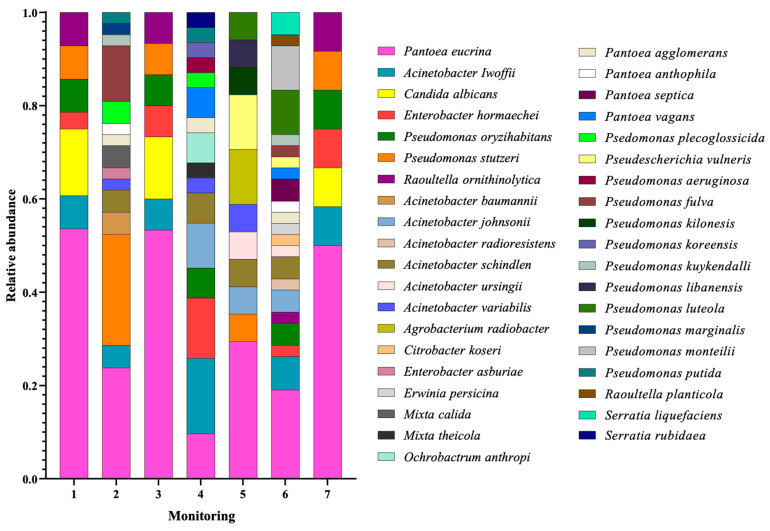
Mass spectrometric identification of Gram-negative bacteria isolated from the gowns of health personnel at Hospital Juárez de México. The results are represented in relative abundance (0 to 1) of the genera and species identified.

The most prevalent genus and species that was consistently identified across all surveys was *P. eucrina* with maximum and minimum relative abundances of 0.57 (survey 1) and 0.56 (survey/area 4) (Figure 2). Interestingly, 3 members of the ESKAPE group were detected, represented by *A. baumannii*, *P. aeruginosa*, and 12 *Enterobacterales*, such as *P. eucrina*, *P. vagans*, *P. agglomerans*, *P. septica*, *P. antophilia*, *E. hormaechei*, *E. asburiae*, *R. ornithinolytica*, *S. rubidaea*, *C. koseri*, and *Erwinia persicina*. Figure 2 shows the results of mass spectrometric identification of Gram-negative bacteria isolated from the gowns of healthcare personnel at HJM.

### 3.5. Flow of ESKAPE Bacteria on Healthcare Staff Gowns by Hospital Service

The distribution of bacteria belonging to the ESKAPE group isolated from the gowns of healthcare personnel across various hospital services was analysed. The bacteria considered in this analysis included *A. baumannii*, *P. aeruginosa*, *Enterobacterales*, and others. The results show clear flow patterns between ESKAPE bacteria and the hospital services where they are most prevalent. The flow diagram revealed that *P. eucrina*, in addition to having the highest frequency of isolation, has a notable flow to all areas analysed, with medical students being the population where the highest number of isolates was identified. However, in the eight critical areas of HJM (adult, neonatal, and paediatric intensive care units, operating theatres, peritoneal dialysis, haemodialysis, pneumology, and toxicology), it was also detected.

In particular, the two *Acinetobacter* species were found to be related to seven of the areas studied, with *A. baumannii* standing out in adult intensive care units, an area where cases of ventilator-associated pneumonia (VAP) are concentrated. Conversely, bacteria such as *E. hormachei* were identified in six hospital services, including the medical student population.

**Figure 3:**
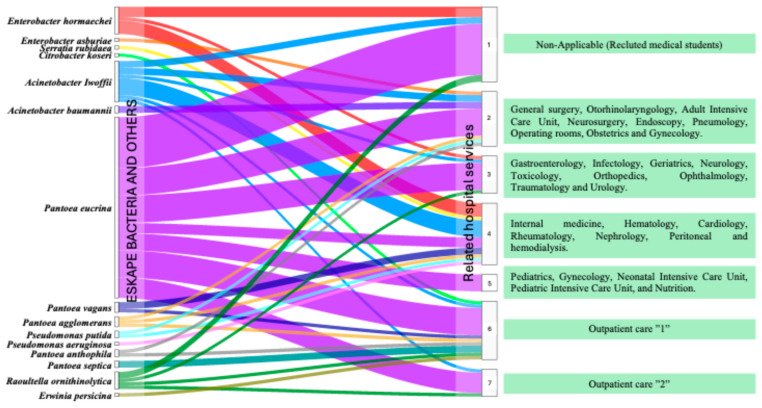
Alluvial analysis of the distribution of ESKAPE bacteria and others isolated from the gowns of healthcare personnel in the different care areas of Hospital Juárez de Mexico.

The results also show the presence of *P. aeruginosa* in area 4. Among other relevant findings, *Pantoea* showed a diverse distribution, occurring in services such as gastroenterology, paediatrics, and general surgery. Regarding outpatient services, other Gram-negative bacteria are more prevalent in outpatient areas (6 and 7) compared to high-specialty hospital services, suggesting external environmental contamination. Figure 3 shows the distribution flow of ESKAPE bacteria and others isolated from healthcare staff gowns in the different care areas of HJM.

### 3.6. Resistance Phenotypes in Isolates from HJM Healthcare Staff Gowns

**Figure 4:**
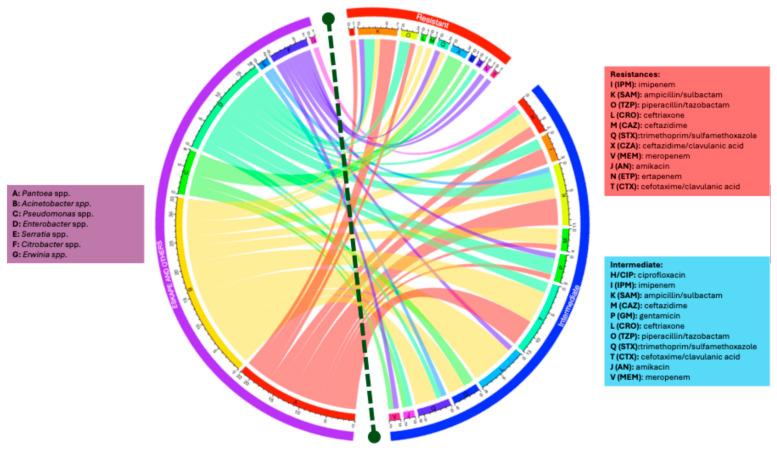
Chord plot analysis of the distribution of antimicrobial resistance phenotypes (resistant and intermediate) in ESKAPE bacteria and others isolated from healthcare workers’ gowns against several antibiotics from different families.

Figure 4 shows the distribution of antimicrobial resistance phenotypes of interest (resistant and intermediate) in ESKAPE bacteria and others isolated from HJM healthcare staff gowns. As it can be observed, the population with the highest burden of antimicrobial “resistance” phenotypes was the *Acinetobacter* group (*A. baumannii* and *A. lwoffii*), represented by β-lactamases, cephalosporins, and folate metabolism inhibitors. Secondly, the genera *Enterobacter* and *Pantoea* showed resistance to the same β-lactamics. Interestingly, the genus *Pseudomonas*, although one of the least frequent resistant groups, showed resistance to carbapenemics (meropenem) and cephalosporins. Finally, the genus *Citrobacter* showed interesting resistance profiles, most notably to trimethoprim/sulfamethoxazole and ertapenem and cefotaxime/clavulanic acid. The genera *Erwinia* and *Serratia* showed profiles categorised as “intermediate resistance”.

### 3.7. Resistance to Cephalosporins and Carbapenemics in ESKAPE Bacteria and Others

The production of BLEE, carbapenemases, and resistance genotypes was assessed in all isolates that showed “resistant and intermediate” categorization profiles to cephalosporins and carbapenems according to CLIS, respectively. This evaluation revealed the presence of carbapenemases and BLEE in nine isolates of *P. eucrina*, where in one isolate the coexistence and/or production of both enzymes was detected. These phenotypes were conferred by serine β-lactamases type *bla_KPC_* and β-lactamase type *bla_CTX-1_* for carbapenemics and cephalosporins, respectively. For *P. putida*, carbapenemase production was identified in one isolate with the *bla_KPC_* genotype. The genotype was not detected in *E. asburiae* and *C. koseri.* Table 3 summarizes the phenotype or carbapenemase and BLEE production findings and the associated genotype according to Table 1.

### 3.8. Analysis of Intergenic Consensus Reveals a Broad Diversity of P. eucrina

**Figure 5:**
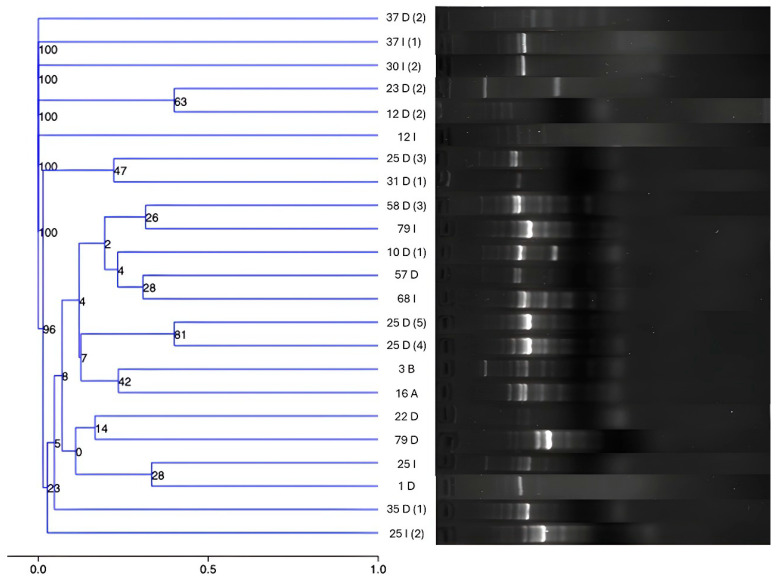
Genomic diversity of *Pantoea eucrina* strains isolated from Hospital Juárez de México health personnel gowns evaluated by dendrogram analysis using ERIC-PCR.

The decision to conduct clonal analysis on *P. eucrina* was driven by its predominance among the isolates recovered from healthcare personnel gowns. Genomic diversity analysis using the ERIC-PCR fingerprinting method revealed sizes of amplicons ranged from slightly more than 100 bp to about 800 bp. Intergenic region diversity of *P. eucrina* showed that the 23 isolates were grouped as unique strains. Therefore, clonal groups were not found. Figure 5 shows the genomic diversity of *P. eucrina* strains isolated from HJM healthcare personnel gowns, as assessed by dendrogram analysis using ERIC-PCR.

### 3.9. Identification of Problems Associated with Microbiological Contamination of Gowns

**Figure 6:**
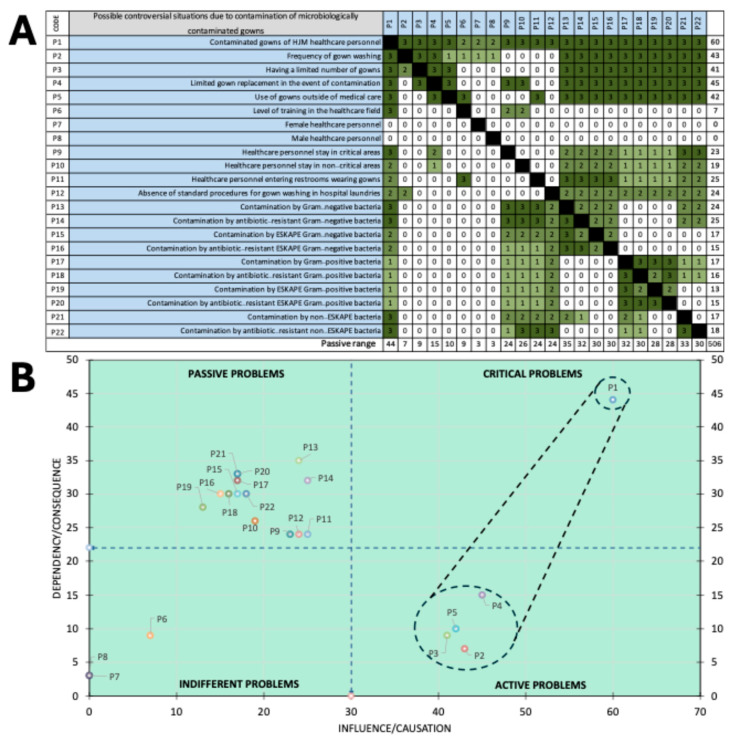
(**A**). Vester matrix constructed with 22 problems due to microbiological contamination of the gowns of health personnel at Hospital Juárez de México. (**B**). Spatial distribution of the 22 problems categorized as passive, critical, indifferent, and active.

Using the microbiological and genetic findings, a Vester matrix was constructed to classify 22 microbiological situations according to their degree of causality to determine which resulted in the four types of microbiological problems associated with the contamination of HJM healthcare staff gowns (Figure 6A). The analysis revealed three indifferent problems (P6 to P9) of low influence and dependence, indicating that the sex of the health personnel and the level of academic training do not represent threats in terms of microbiological contamination of gowns. Thirteen passive problems (P9 to P22) were identified as having a higher dependence but lower influence. These items should be considered for the prevention of contamination of the gowns, although they do not have an immediate impact compared to the critical issues. Finally, four active problems (P2 to P5) were identified as the most significant problems, with high influence and high dependence, indicating that they have a high risk of contributing to microbiological contamination. These problems should be prioritized in interventions to control and prevent contamination of healthcare workers’ gowns.

## 4. Discussion

Classically, the epidemiological triad consists of the pathogen, the host, and the environment, and their interaction is essential in the development of HAIs [35]. Currently, epidemiological research has focused on the study of the agent and the host, but the environment, although often underestimated, has been shown to play a critical role in the chain of pathogen transmission, and its study is key to strengthening infection prevention and control strategies. In previous work by our working group, we have recognized that the “environment” factor includes not only hospital facilities but also medical devices, air, and inert surfaces [18,36,37]. However, a frequently forgotten element is the gown, which, despite being emblematically a symbol of authority and safety in the hospital field, has not been sufficiently considered as a reservoir and vector of pathogens. Therefore, the aim of this study was to assess the health risks associated with microbiologically contaminated gowns of HJM healthcare personnel and to propose infection control strategies focused on this garment, given its potential as a reservoir of antibiotic-resistant pathogens and its role in the transmission of infections. As can be observed in the results section, the microbiological analysis carried out on the gowns of healthcare personnel showed high rates of bacterial contamination by Gram-negative bacteria (61.05%). This finding is relevant, as healthcare staff gowns, which have traditionally been seen as a protective barrier, could be acting as reservoirs of microorganisms, favouring cross-transmission of bacteria between healthcare staff and patients [17,23]. The distribution of bacterial contamination was similar for males and females, indicating that gender did not significantly influence the bacterial load of the gowns (Figure 1B). This result is consistent with previous research that has shown that the gowns of male and female staff are similar [38,39]; however, some others have observed that microbiological contamination rates show an opposite behaviour and reveal higher microbiological contamination related to the sex of healthcare staff [40,41]. Regardless of this variable, these findings reinforce the hypothesis that bacterial contamination on gowns can be sources of transmission, especially when staff do not follow proper gown hygiene and maintenance protocols, including washing [7,8]. Although Gram-positive bacteria such as methicillin-resistant *Staphylococcus aureus* (MRSA) are well-known colonisers in healthcare settings, this study focused on Gram-negative organisms due to their epidemiological predominance at HJM. Surveillance data and prior studies have consistently identified multidrug-resistant *Enterobacterales*, *P. aeruginosa*, and *A. baumannii* as leading pathogens in nosocomial infections and surface contamination [18,20]. Future investigations will explore the role of MRSA and other Gram-positive species in gown-associated transmission.

Additionally, some Gram-negative isolates recovered in this study grouped in *Enterobacterales* were included in the risk analysis. These microorganisms referred to here as “other bacteria” were *Citrobacter koseri*, *Erwinia persicina*, *Mixta*, *Ochrobactrum anthropic*, and others. Despite being less frequent, these organisms exhibited resistance phenotypes to clinically important antibiotics and have been documented in the literature as opportunistic or emerging pathogens in healthcare settings (Table 4). Their detection in critical care areas underscores the importance of including them in the microbiological risk assessment, as they may act as reservoirs of resistance genes and contribute to nosocomial transmission chains.

It is important to note that although no significant difference by sex was observed, it is crucial to identify other variables, such as type of clinical activity and frequency of contact with patients, that could be contributing to contamination. A relevant aspect shown in Figure 1C is the identification of areas where healthcare staff showed higher contamination rates, highlighting areas 2 and 4, where critical patients such as ICU, peritoneal dialysis, and haemodialysis units are admitted (Table 2 and Figure 3). These areas are particularly special because of invasive procedures and the use of medical devices such as mechanical ventilators and catheters in susceptible patients. Interestingly, in one of these areas, *A. baumannii* was identified, a bacterium classified as ESKAPE and a generator of HAIs, with VAP being the most important in our hospital and causing high costs in its care [42].

Regarding bacterial identification by MALDI-TOF mass spectrometry, the technology allowed the identification of a wide diversity of bacterial genera and species (including yeasts such as *C. albicans*). Prevalent genera included *Pseudomonas*, *Acinetobacter*, *Pantoea*, *Enterobacter*, and *Raoultella*, microorganisms that have been directly linked to hospital-acquired infections. A summary of the extensive review of the scientific literature on the microbiological results of this study and their relationship to cases of nosocomial infections is shown in Table 4.

**Table 4:** Summary of infections in patients reported in the scientific literature and compared with the microbiological findings of the present work.

Genus	Species	Healthcare-Associated Infections	Reference
*Pantoea*	*P. eucrina*	Catheter-related bloodstream infection	[25]
		Catheter-related bacteraemia	
		Sepsis	
	*P. agglomerans*	Sepsis	[43]
		Catheter-related bacteraemia	[44]
		Ventilator-associated pneumonia	[45]
		Urinary tract infection	[43]
		Bloodstream infection	[46]
		Soft tissue infection	[43]
	*P. anthophila*	Bloodstream infection	[47]
		Urinary tract infection	[48]
	*P. vagans*	Not reported	Non-applicable
	*P. septica*	Bloodstream infection	[49]
*Acinetobacter*	*A. baumannii*	Ventilator-associated pneumonia	[50]
		Infection of the skin and soft tissues	
		Bacteraemia	
		Catheter-related urinary tract infection	[51]
		Bloodstream Infection	
	*A. Iwoffii*	Catheter-associated bacteraemia	[52]
		Sepsis	[53]
		Pneumonia	
		Urinary tract infection	
		Skin and wound infections	
*Pseudomonas*	*P. putida*	Urinary tract infection	[54]
		Ventilator-associated pneumonia	[55]
		Bacteraemia	[56]
		Bloodstream infection	[57]
		Urinary tract infection	[58]
		Pneumonia	
		Sepsis	
		Wound infection	
	*P. aeruginosa*	Ventilator-associated pneumonia	[50]
		Wound infection	[59]
		Bacteraemia	
		Catheter-related urinary tract infection	[60]
		Bloodstream infection	
		Sepsis	[61]
*Enterobacter*	*E. asburiae*	Ventilator-associated pneumonia	[62]
		Urinary tract infection	
		Sepsis	
		Bacteraemia	[63]
		Infection of the skin and soft tissues	
	*E. hormaechei*	Bacteraemia	[64]
		Ventilator-associated pneumonia	
		Urinary tract infection	
		Sepsis	[65]
		Bloodstream infection	[66]
*Raoultella*	*R. ornithinolytica*	Urinary tract infection	[67]
		Infection of the skin and soft tissues	
		Bacteraemia	
*Ochrobactrum*	*O. anthropi*	Bacteraemia	[68]
		Ventilator-associated pneumonia	
		Sepsis	
		Bloodstream Infection	[69]
*Serratia*	*S. Rubidaea*	Bacteraemia	[70]
		Sepsis	[71]
*Citrobacter*	*C. koseri*	Bacteraemia	[72]
*Erwinia*	*E. persicina*	Not reported	Non-applicable
*Pseudomonas*	*P. stutzeri*	Bacteraemia	[73]
		Bloodstream infection	
		Sepsis	[74]
	*P. fulva*	Bloodstream infection	[57]
		Bacteraemia	
	*P. marginalis*	Not reported	Non-applicable
	*P. kuykendall*	Not reported	Non-applicable
	*P. oryzihabitans*	Catheter-associated bacteraemia	[75]
		Wound infection	
		Ventilator-associated pneumonia	[76]
		Sepsis	[23]
	*P. koreensis*	Not reported	Non-applicable
	*P. luteola*	Ventilator-associated pneumonia	[77]
		Urinary tract infection	[78]
		Bacteraemia	[79,80]
		Bloodstream infection	[81]
	*P. libanensis*	Not reported	Non-applicable
	*P. kilonesis*	Not reported	Non-applicable
	*P. monteilli*	Bacteraemia	[82]
		Sepsis	
*Acinetobacter*	*A. schindleri*	Bacteraemia	[83]
	*A. variabilis*	Not reported	Non-applicable
	*A. johnsonii*	Bloodstream infection	[84]
	*A. ursingii*	Catheter-related bloodstream infection	[85]
		Bacteraemia	[86]
		Sepsis	[87]
	*A. radioresistens*	Bacteraemia	[88]
		Pneumonia	
*Mixta*	*M. calida*	Bacteraemia	[89]
		Sepsis	
	*M. theicola*	Not reported	Non-applicable
*Raoultella*	*R. planticola*	Nosocomial pneumonia	[90]
		Surgical site infection	
		Bloodstream infection	[91]
*Agrobacterium*	*A. radiobacter*	Sepsis	[92]
*Pseudoescherichia*	*P. vulneris*	Not reported	Non-applicable
*Serratia*	*S. liquefaciens*	Bloodstream infection	[93]
		Urinary tract infection	[94]
*Candida*	*C. albicans*	Catheter-related urinary tract infection	[61]
		Bloodstream infection	
		Ventilator-associated pneumonia	

Specifically, *P. eucrina* stood out as the prevalent species, being detected in all areas analysed (Figure 2 and Figure 3). This finding is relevant since, to our knowledge, *P. eucrina* has been little studied in hospital settings, as all efforts in our hospital and elsewhere in the world are focused on the detection and characterisation of typical ESKAPE bacteria [26,95]; however, the presence of *P. eucrina* on staff gowns suggests that it could be playing an important role in the transmission of nosocomial infections. This hypothesis is reinforced because, in previous work in our working group, this microorganism was detected in the linen containers of adult ICUs during COVID-19 [18]. In this study, strain typing was performed exclusively on *Pantoea eucrina* isolates. This decision was based on the fact that *P. eucrina* was the most frequently recovered species and the only one consistently detected across all monitored hospital areas. Its widespread presence suggested the possibility of clonal dissemination or environmental persistence, which warranted deeper analysis. In contrast, other bacterial species were isolated less frequently or appeared in specific areas only, limiting the interpretative value of typing. Thus, the selection of *P. eucrina* for typing was strategic, aiming to understand its potential role as a persistent and underrecognized agent in hospital-associated microbial transmission.

Although *P. eucrina* is not classically categorized as a member of the ESKAPE group, we included it functionally in our analysis due to its consistent isolation across all hospital areas, its phenotypic resistance to critical antibiotics, and its clinical relevance as reflected in Table 4. As a genus within the *Enterobacterales*, *Pantoea* shares phylogenetic and resistance-related features with other established ESKAPE members. The dynamic nature of the ESKAPE framework, as discussed earlier, supports the consideration of non-traditional but increasingly relevant pathogens, especially those with nosocomial potential and antimicrobial resistance as part of an expanded ESKAPE concept. In this context, we propose that *P. eucrina* may be viewed as a potential ESKAPE-related pathogen, particularly in institutions where it demonstrates wide distribution and resistance traits, as shown in our data. This approach aligns with the evolving epidemiology of hospital-acquired infections and the need to adapt bacterial surveillance.

The identification results also showed the presence of bacteria typical of the ESKAPE group, such as *A. baumannii* and *P. aeruginosa*, known for their antibiotic resistance and their ability to cause serious infections in hospitalized patients, especially those with chronic diseases or immunocompromised [96]. On the other hand, *C. albicans* was not the main focus of the present study, its detection on healthcare personnel gowns is noteworthy given its known association with catheter-related infections and others in immunocompromised patients [61]. This incidental finding reinforces the importance of further exploring fungal contamination in future studies. Analysis of bacterial flow through the different areas of the hospital showed that *P. eucrina* had a widespread distribution across all hospital services, including the medical student population (monitoring 1), in addition to having the highest frequency of isolation and showed a remarkable flow towards critical areas (Figure 3). Furthermore, this analysis revealed that medical students had a higher prevalence of this microorganism on their gowns, suggesting that they may act as a vector of transmission (Figure 3). We initially speculated that lack of infection control training was one of the reasons for the high contamination rates. Nevertheless, the Vester matrix classified this situation as an indifferent problem, i.e., with little influence on the main problem. This finding is interpreted on the premise that some basic practices, such as avoiding the use of gowns outside the clinical setting or transferring them to toilets are activities considered obvious, even without formal infection control training. Nonetheless, we speculate that other factors may have a greater impact on gown contamination, such as prolonged unwashed gown use outside the hospital, the absence of institutional gown laundering programs, subjective perceptions of gown cleanliness and personal hygiene habits.

These factors may be more determinant than academic training in infection control. The patterns of antimicrobial resistance phenotypes and genotypes shown in Figure 4 and Table 3 are worrying, as cephalosporins and carbapenemics are the antibiotics used par excellence in therapy at HJM and were the “resistance” and “intermediate resistance” profiles identified in the isolates of the ESKAPE group. This situation compromises and hinders patient recovery from nosocomial infections, especially in immunocompromised patients [97]. The fact that *Pantoea*, *Acinetobacter*, *Pseudomonas*, *Enterobacter*, *Serratia*, *Citrobacter*, and *Erwnia* genera with “resistance” and “intermediate resistance” profiles on healthcare personnel gowns highlights the urgent need for control strategies that not only address the cleaning of surfaces and equipment but also include gown laundering protocols.

A notable finding was the existence and coexistence of carbapenemases and BLEE in *P. eucrina* strains, highlighting their ability to acquire resistance mechanisms to last-line antibiotics. The presence of these enzymes in this microorganism, which to some extent has been forgotten in the hospital environment, is alarming, as it would complicate its elimination in an infectious process. To our knowledge there are few works that have reported this bacterial genus producing *bla_VIM_* and *bla_OXA-48_* carbapenemases and carrying genes associated with the horizontal transfer machinery of extrachromosomal genetic material, which could explain our findings [98,99,100,101]. Finally, the tool for identifying the problems to attack was the Vester matrix.

The fact that four problems were classified as active highlights the urgency of taking measures to reduce microbiological contamination in the gowns of healthcare personnel, especially those in critical areas such as ICUs, operating theatres, and dialysis units, where the risk of transmission could have the greatest impact (Figure 6). Among these, this study identifies actions that may be obvious to some extent but are crucial: increasing the frequency of gown washing, changing gowns in the event of a contamination event (visible or not), and avoiding the use of gowns outside the clinical environment altogether. It is worth noting that, during the time this study was conducted, HJM did not have any formal institutional protocols in place for the management of healthcare personnel gowns. There were no standardized procedures regarding how gowns should be distributed, how often they should be washed, whether they are intended for single or multiple uses, or who is responsible for their maintenance. In practice, most healthcare workers acquire and care for their own gowns individually, often guided by personal habits or perceptions of cleanliness rather than evidence-based practices. This lack of structure creates an environment where the gowns originally intended as a symbol of professionalism and protection can inadvertently become vectors of microbial transmission. Our findings underscore the need to address this gap and to move toward institutional policies that consider gown management as an essential component of infection prevention strategies, especially in critical care settings.

Now, while there is information from other parts of the world where drastic measures have been taken to justify the elimination of gowns for medical staff for the purpose of infection control of *Clostridioides difficile* and methylcillin-resistant *S. aureus* [102,103], there are other studies that have shown that the elimination of gowns could affect the doctor–patient relationship, as well as showing impacts such as the disposal of thousands of white coats and even the “deterioration of the doctor’s image” [104]. Nevertheless, with the rise of multidrug resistance, extensive drug resistance, and pandrug resistance in ESKAPE pathogens, the appraisals of healthcare staff attire are outweighed by the cost–benefit of replacing gowns with short-sleeved uniforms in all hospital services. This hypothesis is reinforced through previous work by our working group, where we demonstrated that VAP can be up to nine times more costly when the causative agent is multidrug-resistant bacteria; not to mention the reduction in mortality [42].

## 5. Conclusions

The findings presented in this study show the importance of implementing infection control strategies that are not limited to the patient and the causative agent. The fact that *P. eucrina* and other medically important microorganisms with antimicrobial resistance are distributed on the gowns of healthcare personnel in training reinforces the idea of the need to improve the importance of this garment, as it has been shown to serve as a significant reservoir of pathogenic bacteria, particularly in critical areas of the hospital. Within our reach, we believe that by increasing the number and frequency of gown laundering, and ideally by having institutional gown laundering programs in place, we could ensure that every attending physician, resident physician, or medical student wears a “safe gown” when in contact with patients, the basis of healthcare personnel’s work.

## Figures and Tables

**Table 1:** Primers used in this study.

Primer	Molecular Target	Sequence (5′→3′)	Size (bp)	Reference
IMP-F	*bla_IMP_*	TTGACACTCCATTTACDG	139	[30]
IMP-R		GATYGAGAATTAAGCCACYCT		
VIM-F	*bla_VIM_*	GATGGTGTTTGGTCGCATA	390	
VIM-R		CGAATGCGCAGCACCAG		
KPC-F	*bla_KPC_*	CATTCAAGGGCTTTCTTGCTGC	538	
KPC-R		ACGACGGCATAGTCATTTGC		
OXA-48F	*bla_OXA-48_*	GCACTTCTTTTGTGATGGC	281	
OXA-48R		GAGCACTTCTTTTGTGATGGC		
SHV-F	*bla_SHV_*	AGCCGCTTGAGCAAATTAAAC	713	
SHV-R		ATCCCGCAGATAAATCACCAC		
CTX-M-1-F	*bla_CTX-1_*	TTAGGAARTGTGCCGCTGYA	688	
CTX-M-1-R		CGATATCGTTGGTGGTRCCAT		
NDM-F	*bla_NDM_*	GGTTTGGCGAT CTGGTTTTC	621	[31]
NDM-R		CGGAATGGCTCATCACGATC		
ERIC1R	Intergenic consensus	ATGTAAGCTCCTGGGGATTCA	Variable	[32]
ERIC2		AGTAAGTGACTGGGGTGAGC		

**Table 2:** Study population categorised according to their function within the Hospital Juárez de México.

Monitoring/Areas		Health Personnel *n* (%)			Sex *n* (%)		Total
		Resident Physicians	Medical Students	Assigned Physicians	Male	Female	
1	NA	0(0)	91(100)	0(0)	41(28.7)	50(28.1)	45(28.3)
2	a	11(45.8)	13(54.2)	0(0)	13(9.1)	11(6.2)	24(7.50)
3	b	31(64.6)	14(29.2)	3(6.3)	21(14.7)	27(15.2)	48(15.0)
4	c	20(66.7)	10(33.3)	0(0)	18(12.6)	12(6.7)	30(9.30)
5	d	28(59.6)	18(38.3)	1(2.1)	13(9.1)	34(19.1)	47(14.60)
6	e	41(85.4)	5(10.4)	2(4.2)	28(19.6)	20(11.2)	48(15.0)
7	f	27(81.8)	5(15.2)	1(3.0)	9(6.3)	24(13.5)	33(10.30)
Totals		157(100)	156(100)	8(100)	143(100)	178(100)	321(100)

a: General surgery, Otorhinolaryngology, Adult Intensive Care Unit, Neurosurgery, Endoscopy, Pneumology, Operating rooms, Obstetrics and Gynecology. b: Gastroenterology, Infectology, Geriatrics, Neurology, Toxicology, Orthopedics, Ophthalmology, Traumatology, and Urology. c: Internal medicine, Hematology, Cardiology, Rheumatology, Nephrology, Peritoneal, and Hemodialysis. d: Pediatrics, Gynecology, Neonatal Intensive Care Unit, Pediatric Intensive Care Unit, and Nutrition. e: Outpatient 1. f: Outpatient 2. NA = Non-Applicable.

**Table 3:** Findings of phenotypes or production of carbapenemases and BLEE and associated genotype in ESKAPE bacteria and others isolated from Hospital Juárez de México healthcare staff gowns.

ESKAPE Bacteria and Others	Phenotype or Producer *n* (%)			Genotype *n* (%)
	Carbapenemases (C)	BLEE (B)	C + BLEE	
*Pantoea eucrina* (*n* = 9)	4 (44.4)	6 (66.6)	1 (11.1)	*bla_KPC_* = 1 (11.1) *bla_CTX-1_* = 2 (22.2)
*Pseudomonas putida* (*n* = 2)	2 (100)	0	0	*bla_KPC_* = 2 (100)
*Enterobacter hormaechei* (*n* = 1)	1 (100)	1	1	ND
*Enterobacter asburiae* (*n* = 1)	1 (100)	0	0	
*Citrobacter koseri* (*n* = 1)	1 (100)	0	0	

ND = Non-Detected.

## Data Availability

Bello-López, Juan Manuel (2025), “Comprehensive Assessment of Health Risks Associated with Gram-Negative Bacterial Contamination on Healthcare Personnel Gowns in Clinical Settings”, Mendeley Data, V1, https://data.mendeley.com/datasets/66vmxc5dxb/1. (accessed on 10 July 2025).
